# C-Reactive Protein for the Early Assessment of Non-Malarial Febrile Patients: A Retrospective Diagnostic Study

**DOI:** 10.3390/diagnostics11091728

**Published:** 2021-09-20

**Authors:** Giulia Bertoli, Cristina Mazzi, Niccolò Ronzoni, Ronaldo Silva, Michele Spinicci, Marco Pozzi, Pietro Sponga, Andrea Aiello, Tamara Ursini, Alessandro Bartoloni, Piero Olliaro, Zeno Bisoffi, Dora Buonfrate

**Affiliations:** 1 Department of Infectious, Tropical Diseases and Microbiology, IRCCS Sacro Cuore Don Calabria Hospital, Negrar di Valpolicella, 37024 Verona, Italy; giulia.bertoli@sacrocuore.it (G.B.); cristina.mazzi@sacrocuore.it (C.M.); niccolo.ronzoni@sacrocuore.it (N.R.); ronaldo.silva@sacrocuore.it (R.S.); tamara.ursini@sacrocuore.it (T.U.); dora.buonfrate@sacrocuore.it (D.B.); 2 Departiment di Medicina Sperimentale e Clinica, Università degli Studi di Firenze, 50121 Firenze, Italy; michele.spinicci@unifi.it (M.S.); pozzim@aou-careggi.toscana.it (M.P.); pietro.sponga@unifi.it (P.S.); andrea.aiello@unifi.it (A.A.); alessandro.bartoloni@unifi.it (A.B.); 3 FIND (Foundation for Innovative Diagnostics), 1202 Geneva, Switzerland; piero.olliaro@ndm.ox.ac.uk; 4 Centre for Tropical Medicine and Global Health, Nuffield Department of Medicine, University of Oxford, Oxford OX3 7LG, UK; 5 Department of Diagnostic and Public Health, University of Verona, 37129 Verona, Italy

**Keywords:** point-of-care tests, non-malarial fever, low- and middle-income countries

## Abstract

Biomarkers, especially CRP, have demonstrated their relevance to differentiate viral from bacterial infection, even though a reliable threshold is far to being found. In low- and middle-income countries, affordable and user-friendly rapid diagnostic tests based on biomarkers can be widely adopted to help health workers in the management of non-malarial fever. The primary objective of this study is to assess the best CRP cut-off to distinguish viral from bacterial infections. Other biomarkers were evaluated for the same purpose, alone or in combination with CRP. We retrospectively collected data from two referral hospital departments for infectious and tropical diseases in Italy. Areas under the ROC curve (AUC) were calculated and then compared using the DeLong test. Overall, we included 1193 febrile cases (viral 20.74% vs. bacterial 79.25%). We also collected malaria (*n* = 202) and intestinal parasite (*n* = 186) cases to establish their impact on biomarkers. CRP had the best accuracy in differentiating viral from bacterial infections. The best performance of CRP was a cut-off of 11 mg/L. All other biomarkers studied had significantly lower accuracy. Median CRP values were within the normal ranges in parasitic infections, while they were higher in malaria. None of the combinations of CRP with other biomarkers significantly increased the accuracy of CRP alone.

## 1. Introduction

Antibiotic-resistant bacteria are an increasing public health concern worldwide [1]. This trend is largely attributable to overtreatment, misdiagnosis and mismanagement of acute febrile infections. The just-in-case prescription of antibiotics is a generalized practice due to the lack of point-of-care diagnostics, especially in settings like primary health care centres of low- to middle-income countries (LMIC), where laboratory support is missing [2].

In malaria-endemic countries, the deployment of malaria rapid diagnostic tests (RDT), according to the WHO test-and-treat recommendation for febrile patients [3], resulted in better targeted use of antimalarial drugs overall, despite differences between endemic regions [4]. However, once malaria is excluded, proper management of febrile patients remains problematic. Practical tools such as the Integrated Management of Childhood Illnesses (IMCI) manual are a valid support for the management of febrile children with malaria-positive RDT, but include less robust evidence for workup and management of malaria-negative cases [5], generally resulting in unrestrained use of antibiotics even for viral infections [1,6,7].

With few available pathogen-specific point-of-care diagnostic tests, biomarkers might help differentiate viral from bacterial malaria-negative fevers and guide the prescription of antibiotics. In high-income countries (HIC), biomarkers are variably used for the management of febrile inpatients, both for clinical assessment and for monitoring. However, the usefulness of this approach has an incomplete base of evidence, mostly based on childhood respiratory infections [8]. In addition, in LMIC, cut-off values may be variably influenced by co-morbidities such as malaria, HIV, malnutrition, and intestinal parasites [9].

Studies evaluating the use of biomarkers in non-malarial febrile patients in LMIC [10] are heterogeneous in terms of geographical region, populations, and diagnostic approach. Although these discrepancies make estimates of the accuracy of specific biomarkers difficult to generalize, C-reactive protein (CRP) is one of the most promising biomarkers for LMIC. CRP is widely used as a marker of inflammation and infection in routine practice. Different devices have been implemented for its detection and quantification, including point-of-care tests that can be suitable for LMIC [11]. Overall, a better understanding of CRP’s role in early diagnosis of common febrile tropical diseases, especially in differentiating viral and bacterial causes, may inform antibiotic guidance strategies, with a potential favourable impact on antimicrobial stewardship and on reducing drug pressure and antibiotic resistance.

In this study, carried out in two referral hospital departments for infectious and tropical diseases in Italy, we aimed to investigate the utility of CRP as a marker in the differential diagnosis of common febrile infectious diseases, in particular to discriminate viral infections, for which no specific treatment is available/warranted, from bacterial causes of fever, which would require antibiotic treatment. The primary objective was to assess the best CRP cut-off to distinguish a viral from a bacterial infection; the secondary objective was to evaluate other biomarkers for the same purpose.

## 2. Materials and Methods

### 2.1. Study Population and Setting

We retrospectively collected data from medical records of adult inpatients, admitted to the Department of Infectious-Tropical Diseases and Microbiology (DITM), IRCCS Sacro Cuore Don Calabria Hospital, Negrar di Valpolicella, Verona (Italy), and to the Infectious and Tropical Diseases Unit, Careggi University Hospital, Firenze (Italy), between January 2008 and December 2018.

Inclusion criteria:

Febrile patients aged ≥ 18 years presenting one of the infectious conditions listed in Table 1.

Exclusion criteria:

- Patients who did not fulfil the diagnostic criteria.
- Records with incomplete demographic data.
- Records with no CRP value available within 48 h of the initiation of specific treatment.

From each medical record, we retrieved the demographic and clinical characteristics described in Supplementary Materials Table S1. Laboratory data included CRP, white blood cells (WBC), lactate dehydrogenase (LDH), fibrinogen, and erythrocyte sedimentation rate (ESR).

### 2.2. Disease Group Classification

The conditions listed in Table 1 were grouped into the following 4 main groups:

1. Viral infections (including viral meningitis, arbovirus infections and flu);
2. Bacterial infections, including blood stream infections, bacterial meningitis, bacterial pneumonia, zoonotic infections (Brucella, Leptospira), and complicated flu with secondary bacterial infections likely requiring antibiotic treatment;
3. Malaria;
4. Intestinal parasites and schistosomiasis.

Analyses to determine CRP accuracy in differentiating between infections requiring or not requiring antibiotics were performed on groups 1 and 2. The other groups were included to evaluate how co-infections frequently found in LMIC affect CRP values and thus its discriminatory capacity.

### 2.3. Data Management and Confidentiality

Data were collected into a standardized electronic case report form (CRF) set up on Open Clinica. Five investigators entered the data, and the principal investigator supervised the data entry. Each participating site entered data starting from the most recent cases (by date of admission), selecting eligible patients until the specified sample size limit for a given infection was reached (see below).

Under the EU General Data Protection Regulation (GDPR) 2016/679, patients’ sensitive data were not collected. Each observation in the data set was identified by an alphanumerical code not related to patient identification. The data manager monitored on a regular basis the data quality checks and performed related tasks such as data cleaning. The data quality procedure included monitoring the validity, accuracy, completeness, consistency, and uniformity of the collected data.

Primary outcome: Sensitivity and specificity of CRP to differentiate viral from bacterial causes of non-malarial febrile illness (NMFI).

Secondary outcomes: Sensitivity and specificity of other selected biomarkers, alone or in combination with CRP, to differentiate viral from bacterial causes of NMFI. Biomarkers considered were WBC, neutrophils, fibrinogen, leucocytes, eosinophils, and LDH.

### 2.4. Sampling and Sample Size

Sampling was based on convenience criteria (mainly the availability of cases). The records of all patients fulfilling the inclusion criteria during the study period were included, with the only limitation of stopping the inclusion of a given, single diagnosis once a maximum number of 250 was reached (to avoid some conditions being over-represented).

### 2.5. Statistical Analysis

Descriptive statistics were calculated for demographic and clinical data in relation to the different diagnoses. In Florence, CRP values were reported by the laboratory on a continuous scale starting from 8 mg/dL, while values reported by the laboratory in Negrar were on a scale starting from 0 mg/dL. For data harmonization purposes, CRP levels lower than 8 were reassigned to CRP = 8. Generalized linear mixed models (GLMM) were used to test the association of viral and bacterial diseases with CRP and other relevant clinical biomarkers. Biomarkers with a percentage of missing values higher than 25% were not included. Cut-off analysis maximizing the percentage of correctly classified cases was performed only for the biomarkers that could significantly discriminate between viral and bacterial infections (*p* ≤ 0.05) (Supplementary Materials Table S2). Cut-off points with their sensitivity and specificity were reported along with their 95% confidence interval (CI). Areas under the ROC curve (AUC) were calculated and then compared using the DeLong test. Data were analysed using SAS (version 9.4; SAS Institute Inc., Cary, NC, USA).

### 2.6. Ethical Approval

The study protocol obtained ethical clearance from the “Comitato Etico per le province di Verona e Rovigo” on 6 March 2019 (protocol number 14756).

## 3. Results

### 3.1. Demographic, Clinical, and Laboratory Characteristics (Viral vs. Bacterial)

**Figure 1:**
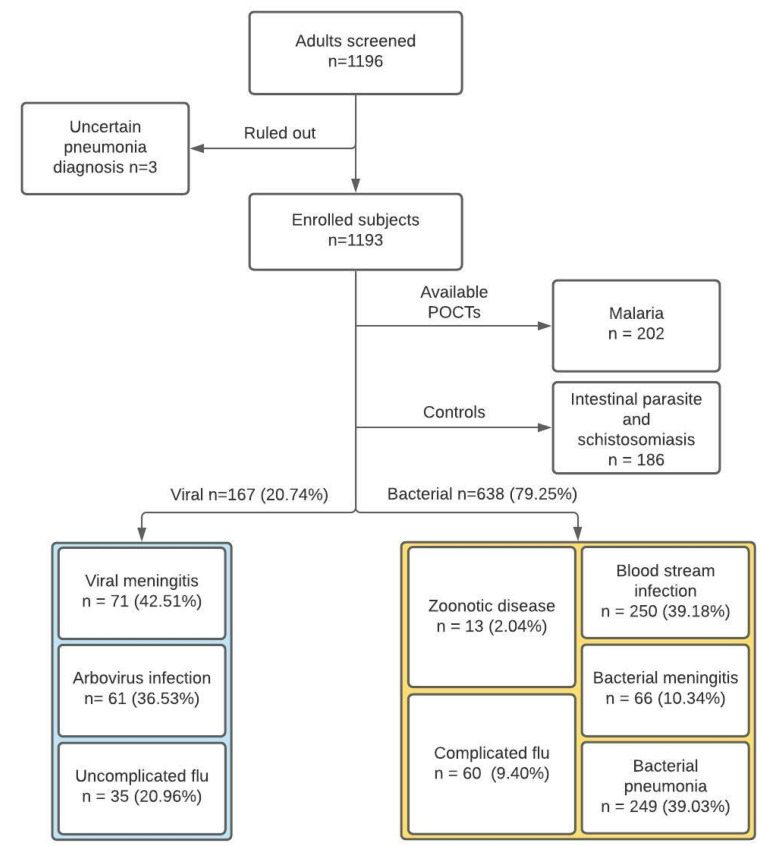
Study flow chart.

The study flow is summarized in Figure 1.

Overall, we included 167 and 638 cases of viral and bacterial NMFI, respectively, 202 cases of malaria, and 186 cases of non-febrile parasitic infections.

The number of cases contributed by the study sites is reported in Table 2.

The main demographic and clinical characteristics of the patients included are reported in Table 3.

### 3.2. Biomarkers (Viral vs. Bacterial)

**Figure 2:**
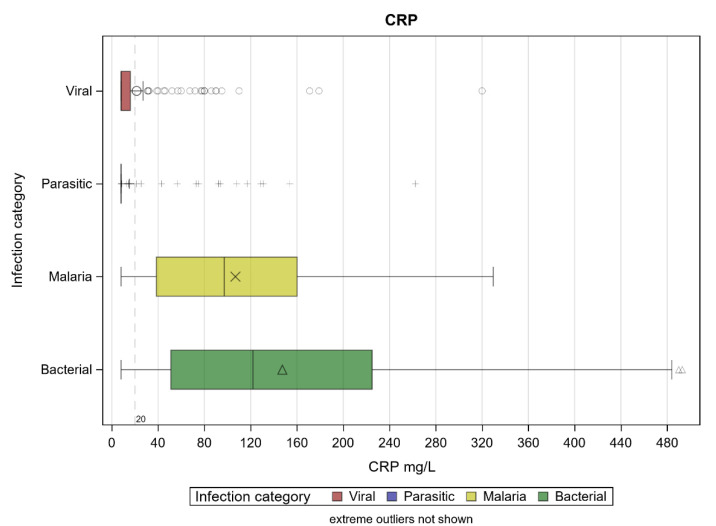
CRP levels according to infection category. Extreme outliers are not shown.

The median CRP values of viral and bacterial infections were 8.00 (IQR: 8.00–16.00) and 122 (IQR: 51.00–225.00) mg/L, respectively (*p* < 0.001) (Figure 2).

Median CRP values were within the normal ranges in parasitic infections, while they were higher in malaria (97.20, IQR: 38.49–160.09), although they were significantly lower than those found for bacterial infections (*p* < 0.0001). The median values (and corresponding interquartile ranges) of the other biomarkers, in relation to the groups of infections, are reported in Table 4.

**Figure 3:**
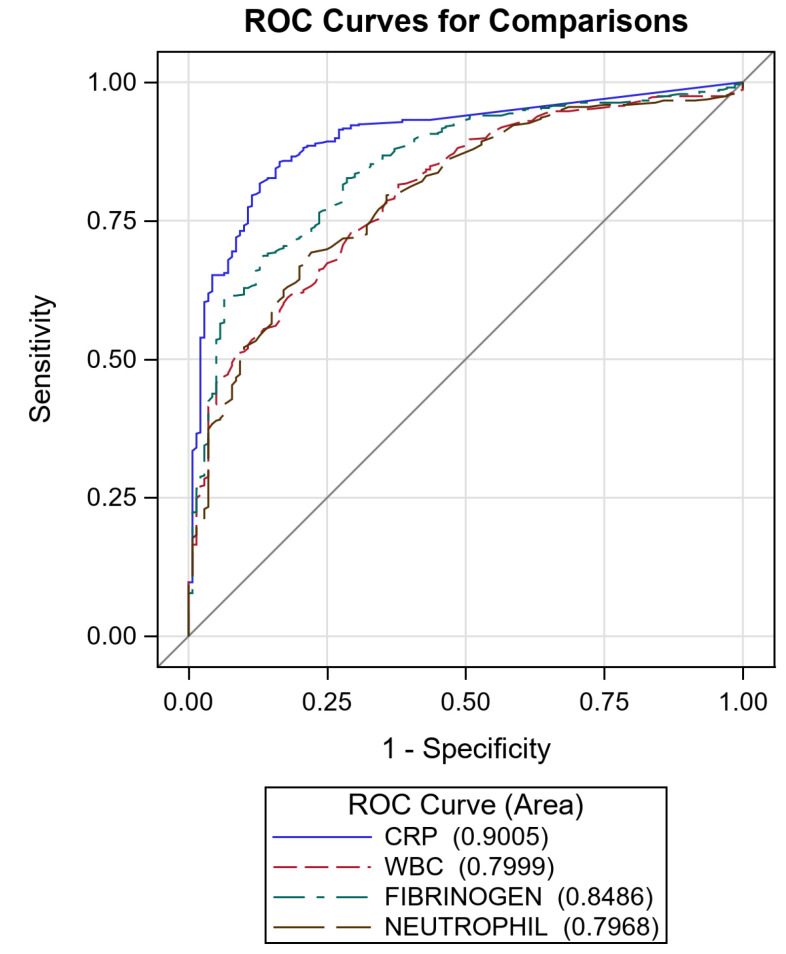
Receiver Operating Characteristic curves of C-reactive protein (CRP) (AUC 90.0%, 95% CI: 87.4–92.7), fibrinogen (AUC 84.9%, 95% CI: 81.4–88.3), white blood cells (WBC) (AUC 80.0%, 95% CI: 76.1–83.9), and neutrophil (AUC 79.7%, 95% CI: 75.7–83.6). Diagonal line represents line of reference.

ERS was excluded from further analysis due to the high missing rate. For the other markers, including CRP, ROC curves were created (Figure 3).

As can be observed from the AUC, CRP showed the best accuracy, followed by fibrinogen. The accuracy of CRP was statistically different from all other markers (*p* < 0.0001 for CRP versus WBC and versus neutrophils, *p* = 0.0009 for CRP versus fibrinogen). The cut-off values that allowed for the best accuracy were calculated through the ROC curves and are reported in Table 5.

**Figure 4:**
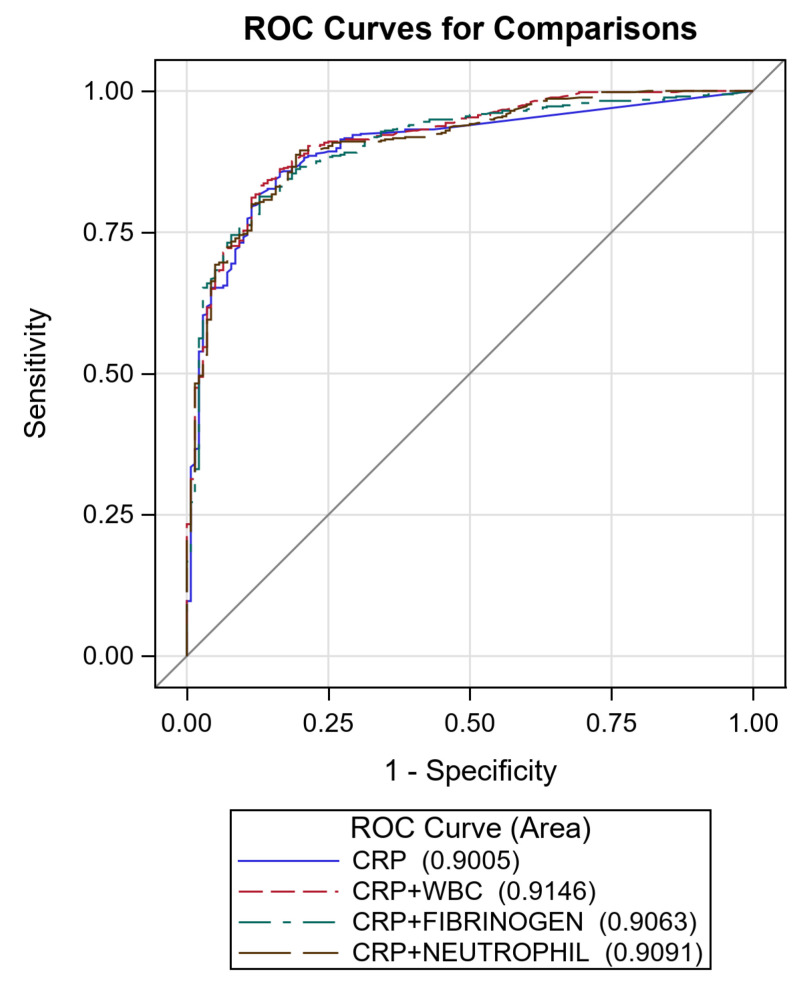
Receiver Operating Characteristic curves of C-reactive protein (CRP) + white blood cells (WBC) (AUC 91.5%, 95% CI: 89.0–93.9), CRP + fibrinogen (AUC 90.6%, 95% CI: 88.0–93.3) and CRP + neutrophil (AUC 90.9%, 95% CI: 88.4–93.5). Diagonal line represents line of reference.

As an exploratory analysis, we evaluated the accuracy of the combination of CRP plus the other biomarkers against CRP alone (Figure 4). None of the combinations significantly increased the accuracy of CRP alone.

## 4. Discussion

In this study, we found that, among the selected biomarkers, CRP had the best accuracy in differentiating viral from bacterial infections. CRP’s best performance was with a cut-off of 11 mg/L. All other biomarkers studied had significantly lower accuracy, with specificities that were particularly low (under 50%) when the corresponding sensitivity was deemed sufficiently good (>92%) to avoid missing a substantial proportion of bacterial infections. We also evaluated the combination of CRP with the other biomarkers, but none of them appeared to increase the accuracy of CRP alone. Parasitic infections, which are frequently found in LMIC, did not influence CRP levels; hence there would be no need to rule out these infections to interpret the results of CRP. On the other hand, in accordance with previous evidence [7,12], malaria increased CRP, though to levels that were significantly lower than those found in bacterial infections. Malaria RDTs are systematically used in acute febrile illnesses in malaria-endemic countries. While a negative malaria RDT would then support the prescription of antibiotics in a febrile patient with high CRP, a positive malaria RDT test would still leave the healthcare provider with their clinical judgement, as one cannot rule out with all certainty a concomitant bacterial infection.

Selecting a cut-off value is a trade-off between minimising false-negatives (high sensitivity) vs. false-positives (specificity). Erring on the side of caution to minimise the risk of missing cases that require antibiotics means that a relatively low cut-off value (like the 11 mg/L found here) may not have a huge impact on antibiotic prescriptions. Our findings are in line with a recent meta-analysis and individual patient data study [13], which showed that a CRP cut-off close to the one found here (10 mg/L) achieved very high sensitivity for bacterial infections. However, the optimal accuracy was found at 36 mg/L, and at a threshold of 40 mg/L the sensitivity and specificity for bacterial infections were 74% (95% CI 70–77) and 84% (95% CI 81–87), respectively. The combined use of the two cut-offs was evaluated for routine care and proved useful to significantly increase the correct classification of the NMFI, although cases with CRP <40 and >10 mg/L could not be classified. On the other hand, a previous study showed that CRP at a cut-off of 10 mg/L had high sensitivity for the detection of bacterial infections (95%, 95% CI 92–97), while specificity was extremely low (49%, 95% CI 46–53) [7].

Other studies previously assessed different CRP cut-off values, trying to find a good compromise between sensitivity and specificity. However, it has not yet been possible to identify an optimal cut-off at which CRP would meet the target product profile proposed (sensitivity ≥90% and specificity ≥80% for differentiating bacterial from non-bacterial infections in children with non-severe, non-malarial acute fever in low-resource settings) and the ASSURED criteria [9]. It must be considered that differences in settings and age of the patient population in question could hamper the generalization of the findings. Finally, the impact on clinical outcome/antibiotic prescription of a CRP-based approach of NMFI in LMIC has yet to be defined, with the few available studies producing inconsistent results.

The utility of CRP would likely improve when combined with other diagnostic tools and/or clinical algorithms, as has been suggested [5,14], especially if using point-of-care, rapid diagnostic tests [15].

This study is not without limitations. First, the study sites are not in LMIC—settings the results are mainly intended for. However, a strength of the study is that it allowed for well-characterized infections, thanks to the availability of specific laboratory tests and imaging, which are often missing in studies conducted in LMCI [16]. Second, different CRP scales were used in the two study sites. While the values were then harmonized, a continuous scale including values <8 might have been more precise; however, this did not seem to affect the calculation of the cut-off values of CRP and the estimation of its accuracy. Another strength of the study is the inclusion of individuals of 18 years of age and older. Very few studies have focused on adults with NMFI [10]; hence we believe that our findings can add valuable data for this age group.

## 5. Conclusions

In conclusion, among the biomarkers evaluated, CRP showed the best accuracy in differentiating viral from bacterial infections. A CRP cut-off value of 11 mg/L would miss a small proportion of bacterial infections, while also leaving an unsatisfactorily high proportion of viral infections wrongly classified and hence probably mismanaged with antibiotics. Combining CRP with other biomarkers does not increase accuracy significantly. In line with previous evidence, our study supports the implementation and evaluation of CRP-based devices in combination with other diagnostic tools and clinical algorithms, for the clinical management of NMFI in LMIC.

## Figures and Tables

**Table 1:** Causes of fever and diagnostics.

Infectious Disease	Diagnostic Criteria	Type of Infection
MALARIA	Parasitological identification, PCR	Not viral
BLOOD STREAM INFECTION	Microbiological identification	Not viral
BACTERIAL PNEUMONIA	Radiological signs, microbiological identification, urinary antigens	Not viral
BACTERIAL MENINGITIS (*S. pneumoniae*, *N. meningitidis*, *H. influenzae*)	Microbiological identification, PCR	Not viral
ZOONOTIC DISEASES (brucellosis, leptospirosis, rickettsiosis)	Serology, PCR	Not viral
UNCOMPLICATED FLU	Serology, PCR	Viral
COMPLICATED FLU (OVER INFECTION)	Serology, PCR	Not viral
ARBOVIRUS INFECTION (dengue, chikungunya, zika)	Serology, PCR	Viral
VIRAL MENINGITIS	Serology, PCR	Viral
INTESTINAL PARASITE INFECTION AND SCHISTOSOMIASIS	Serology, parasitological identification, PCR	Controls

**Table 2:** Cases from the study site.

Center	Viral N (%)	Bacterial N (%)	Parasitic N (%)	Malaria N (%)	Overall N (%)
FIRENZE	131 (78.4)	405 (63.5)	32 (17.2)	57 (28.2)	625 (52.4)
NEGRAR	36 (21.6)	233 (36.5)	154 (82.8)	145 (71.8)	568 (47.6)
Overall	167	638	186	202	1193

**Table 3:** Main characteristics of the included patients.

Demographical Data	N	Overall	Diagnoses			
			Viral	Bacterial	Parasitic	Malaria
FEMALE, N (%)	1193	434 (36.4)	77 (46.1)	269 (42.1)	30 (16.1)	58 (28.7)
AGE (YEARS), MEDIAN (IQR)	1193	46 (32–63)	37 (30–50)	57 (42–69)	25 (21–34)	41 (33–52)
PATIENTS’ ORIGIN, N (%)	1193					
ITALY		709 (59.4)	125 (74.9)	561 (65.8)	15 (8.1)	60 (29.7)
AFRICA		343 (28.8)	7 (4.2)	140 (16.4)	157 (84.4)	128 (63.4)
SOUTHEAST ASIA		27 (2.3)	6 (3.6)	42 (4.9)	6 (3.2)	3 (1.5)
OTHER		114 (9.6)	29 (17.4)	110 (12.9)	8 (4.3)	11 (5. 4)
CLINICAL DATA						
HIV, N (%)	1193	40 (4.2)	7 (3.8)	24 (4.4)	1 (0.5)	8 (4.0)
FEVER ON ADMISSION, N (%) TEMPERATURE ON ADMISSION, MEDIAN (IQR)	1171 478	485 (41.4) 38.30 °C (37.90–38.90)	90 (53.9) 38.00 °C (37.80–38.50)	301 (47.6) 38.40 °C (38.00–39.00)	4 (2.3) 37.75 °C (37.65–38.15)	90 (46.0) 38.40 °C (38.00–39.00)
FEVER IN THE LAST 24 HOURS, N (%) TEMPERATURE IN THE LAST 24 HOURS, MEDIAN (IQR)	1190 391	677 (56.0) 39.00 °C (38.30–39.30)	110 (65.9) 39.00 °C (38.05–39.00)	419 (65.7) 39.00 °C (38.50–39.50)	3 (1.6) 40.00 °C (40.00–40.00)	145 (71.8) 39.00 °C (38.00–39.10)
MAXIMUM OBSERVED TEMPERATURE DURING HOSPITALIZATION, MEDIAN (IQR)	1108	38.00 °C (37.00–39.00)	38.00 °C (37.50–38.70)	38.40 °C (37.50–39.00)	36.50 °C (36.20–36.70)	38.30 °C (37.00–39.20)
FEVER DURATION (DAYS), MEDIAN (IQR)	1113	2.00 (0.00–5.00)	3.00 (1.00–5.00)	3.00 (1.00–5.00)	0.00 (0.00–0.00)	1.00 (0.00–2.00)
CARDIAC FREQUENCY (BPM), MEDIAN (IQR)	1090	82.00 (72.00–95.00)	80.00 (70.00–89.00)	87.00 (76.00–100.00)	70.00 (60.00–80.00)	83.5 (72.00–98.00)
DIED, N (%)	1193	13 (2.0)	0 (0.0)	13 (2.0)	0 (0.0)	0 (0.0)

NOTE. N is the number of non-missing values. ABBREVIATIONS. IQR: Interquartile Range, BPM: Beats Per Minute.

**Table 4:** Median values of other biomarkers.

Biomarkers Median (IQR)	N	Overall	Diagnoses			
			Viral	Bacterial	Parasitic	Malaria
WBC (N/µL)	1190	6.80 (4.50–11.00)	4.70 (3.10–7.20)	8.60 (6.00–13.50)	5.30 (4.10–6.40)	4.90 (4.00–6.10)
Neutrophils (N/µL)	1131	4.10 (2.20–7.80)	2.80 (1.40–4.80)	7.10 (4.30–11.80)	2.05 (1.5–2.80)	2.90 (1.80–4.00)
Fibrinogen (mg/dL)	1044	4.79 (3.51–6.19)	3.88 (3.22–4.78)	5.82 (4.83–7.90)	2.49 (2.25–3.01)	4.17 (3.51–4.98)
ESR (mm/h)	734	30.00 (12.00–54.00)	14.00 (8.00–24.50)	52.00 (35.00–67.00)	9.00 (6.00–16.00)	30.00 (15.00–52.00)

NOTE. N is the number of valid cases. WBC: White Blood Cells, CRP: C-Reactive Protein, ESR: Erythrocyte Sedimentation Rate, IQR: Interquartile Range.

**Table 5:** Biomarkers’ cut-offs with corresponding sensitivity and specificity. Cut-offs were obtained maximizing the percentage of correctly classified cases.

Biomarkers	Cut-Off	Sensitivity (95% CI)	Specificity (95% CI)
CRP	11.00	92.8 (90.8–94.8)	69.3 (62.3–76.3)
WBC	3.60	93.7 (91.8–95.6)	32.3 (25.2–39.4)
FIBRINOGEN	3.85	93.5 (91.4–95.5)	48.3 (40.1–56.4)
NEUTROPHILS	1.60	95.1 (93.3–96.8)	29.2 (22.2–36.2)

CRP: C-Reactive Protein, WBC: White Blood Cells, CI: Confidence Interval.

## Data Availability

Raw data are going to be available on Mendeley Data.

## Supplementary Materials

The following are available online at https://www.mdpi.com/article/10.3390/diagnostics11091728/s1, Table S1: Demographical and clinical characteristics, Table S2: Clinical biomarkers associated with bacterial diseases.

## References

[1] World Health Organization (2014). Antimicrobial Resistance: Global Report on Surveillance 2014.

[2] World Health Organization (2013). WHO Informal Consultation on Fever Management in Peripheral Health Care Settings: A global Review of Evidence and Practice.

[3] World Health Organization (2015). Guidelines for the Treatment of Malaria.

[4] Bisoffi Z., Sirima S.B., Menten J., Pattaro C., Angheben A., Gobbi F., Tinto H., Lodesani C., Neya B., Gobbo M. (2010). Accuracy of a rapid diagnostic test on the diagnosis of malaria infection and of malaria-attributable fever during low and high transmission season in Burkina Faso. Malar. J..

[5] Keitel K., Kagoro F., Samaka J., Masimba J., Said Z., Temba H., Mlaganile T., Sangu W., Rambaud-Althaus C., Gervaix A. (2017). A novel electronic algorithm using host biomarker point-of-care tests for the management of febrile illnesses in Tanzanian children (e-POCT): A randomized, controlled non-inferiority trial. PLoS Med..

[6] Hildenwall H., Muro F., Jansson J., Mtove G., Reyburn H., Amos B. (2017). Point-of-care assessment of C-reactive protein and white blood cell count to identify bacterial aetiologies in malaria-negative paediatric fevers in Tanzania. Trop. Med. Int. Health TM IH.

[7] Lubell Y., Blacksell S.D., Dunachie S., Tanganuchitcharnchai A., Althaus T., Watthanaworawit W., Paris D.H., Mayxay M., Peto T.J., Dondorp A.M. (2015). Performance of C-reactive protein and procalcitonin to distinguish viral from bacterial and malarial causes of fever in Southeast Asia. BMC Infect. Dis..

[8] Aabenhus R., Jensen J., Jørgensen K., Hróbjartsson A., Bjerrum L. (2014). Biomarkers as point-of-care tests to guide prescription of antibiotics in patients with acute respiratory infections in primary care. Cochrane Database Syst. Rev..

[9] Escadafal C., Nsanzabana C., Archer J., Chihota V., Rodriguez W., Dittrich S. (2017). New Biomarkers and Diagnostic Tools for the Management of Fever in Low- and Middle-Income Countries: An Overview of the Challenges. Diagnostics.

[10] Bertoli G., Ronzoni N., Silva R., Spinicci M., Perlini C., Omega L., Ursini T., Bartoloni A., Olliaro P., Bisoffi Z. (2020). Usefulness of C-Reactive Protein and Other Host BioMarker Point-of-Care Tests in the Assessment of Non-Malarial Acute Febrile Illnesses: A Systematic Review with Meta-Analysis. Am. J. Trop. Med. Hyg..

[11] Vashist S.K., Venkatesh A.G., Schneider E.M., Beaudoin C., Luppa P.B., Luong J.H.T. (2016). Bioanalytical advances in assays for C- reactive protein. Biotechnol. Adv..

[12] Kortz T.B., Nyirenda J., Tembo D., Elfving K., Baltzell K., Bandawe G., Rosenthal P.J., Macfarlane S.B., Mandala W., Nyirenda T.S. (2019). Distinct Biomarker Profiles Distinguish Malawian Children with Malarial and Non-malarial Sepsis. Am. J. Trop. Med. Hyg..

[13] Otten T., de Mast Q., Koeneman B., Althaus T., Lubell Y., van der Ven A. (2021). Value of C-reactive protein in differentiating viral from bacterial aetiologies in patients with non-malaria acute undifferentiated fever in tropical areas: A meta-analysis and individual patient data study. Trans. R. Soc. Trop. Med. Hyg..

[14] Kapasi A.J., Dittrich S., Gonzalez I.J., Rodwell T.C. (2016). Host Biomarkers for Distinguishing Bacterial from Non-Bacterial Causes of Acute Febrile Illness: A Comprehensive Review. PLoS ONE.

[15] Salami O., Horgan P., Moore C.E., Giri A., Sserwanga A., Pathak A., Basnyat B., Kiemde F., Smithuis F., Kitutu F. (2020). Impact of a package of diagnostic tools, clinical algorithm, and training and communication on outpatient acute fever case management in low- and middle-income countries: Protocol for a randomized controlled trial. Trials.

[16] Althaus T., Lubell Y., Maro V.P., Mmbaga B.T., Lwezaula B., Halleux C., Biggs H.M., Galloway R.L., Stoddard R.A., Perniciaro J.L. (2020). Sensitivity of C-reactive protein for the identification of patients with laboratory-confirmed bacterial infections in northern Tanzania. Trop. Med. Int. Health.

