# Supplementary material for: C-Reactive Protein for the Early Assessment of Non-Malarial Febrile Patients: A Retrospective Diagnostic Study
Source: Diagnostics (Basel). 2021 Sep 20;11(9):1728. doi: 10.3390/diagnostics11091728 (PMC8469932; doi:10.3390/diagnostics11091728)
Supplement: Supplementary file 1 [file diagnostics-11-01728-s001.zip › diagnostics-1386299-supplementary.pdf]

**Supplementary Table S1.** Demographical and clinical characteristics

| | |
|---|---|
| **DEMOGRAPHICAL DATA** | |
| SEX | (FEMALE/MALE) |
| AGE > 18 | (YEARS) |
| PATIENTS' ORIGIN | |
| **CLINICAL DATA** | |
| HIV | (YES/NO) |
| FEVER ON ADMISSION<br>TEMPERATURE ON ADMISSION | (YES/NO)<br>(ºC) |
| FEVER IN THE LAST 24 HOURS<br>TEMPERATURE IN THE LAST 24 HOURS | (YES/NO)<br>(ºC) |
| MAXIMUM OBSERVED TEMPERATURE DURING HOSPITALIZATION | (ºC) |
| FEVER DURATION | (DAYS) |
| CARDIAC FEQUENCY | (bpm) |
| DIAGNOSIS<br>DIAGNOSE CONFIRMED | ICD9*<br>Serology, parasitological/histological/microbiological identification, PCR, radiological signs, urinary antigens |
| DIED | (YES/NO) |

* International classification of Diseases

**Supplementary Table S2**. Clinical biomarkers associated with bacterial diseases, results of univariable logistic regressions.

| BIOMARKER | N | OR | 95% CI | P-value |
|---|---|---|---|---|
| CRP | 804 | 1.037 | 1.030 - 1.045 | <.0001 |
| WBC | 804 | 1.295 | 1.227 - 1.367 | <.0001 |
| NEUTROPHILS | 748 | 1.370 | 1.280 - 1.466 | <.0001 |
| LEUCOCYTES | 750 | 0.967 | 0.820 - 1.141 | 0.6930 |
| EOSINOPHILS | 697 | 4.520 | 0.727 - 28.096 | 0.1055 |
| LDH | 657 | 1.001 | 0.999 - 1.002 | 0.3654 |
| FIBRINOGEN | 695 | 2.809 | 2.279 - 3.463 | <.0001 |

NOTE. CRP: N is the number of non missing values. C-Reactive Protein. WBC: White Blood Cells. LDH: Lactate Dehydrogenase. OR: Odds Ratio, CI: Confidence Intervals.
